# The association of nitrate exposure with bone mineral density in adolescents aged 12 to 19: A cross-sectional study

**DOI:** 10.1097/MD.0000000000048351

**Published:** 2026-04-17

**Authors:** Shuo Duan, Shuaishuai Wang, Zhiyang Liu, Shuaiwei Li, Yisi Wang, Minglei Zhang

**Affiliations:** a Department of Orthopedics, China-Japan Union Hospital, Jilin University, Changchun, Jilin, China.

**Keywords:** adolescents, bone mineral density, NHANES, nitrate, public health

## Abstract

The association between urinary nitrates exposure and bone mineral density (BMD) in adolescents is unclear. This study aimed to examine the association between nitrate levels and BMD in adolescents aged 12 to 19. Cross-sectional data were obtained from the National Health and Nutrition Examination Survey conducted between 2011 and 2018. Weighted multivariate logistic regression analyses, and sensitivity analyses were employed to assess the independent association between BMD and nitrate levels, with subgroup analyses based on sex, age, poverty-to-income ratio and race. Additionally, smooth curve fitting and saturation threshold analysis were employed to explore nonlinear relationships. After adjusting for relevant covariates, nitrate levels were found to be negatively associated with lumbar spine BMD (β = −0.036, 95% confidence interval: −0.054, −0.019) and total BMD (β = −0.027, 95% confidence interval: −0.040, −0.014). Subgroup analyses demonstrated that this negative correlation remained consistent across all subgroups. Smoothed curve fitting also confirmed a negative relationship between nitrate levels and BMD. The nonlinear relationship between nitrate levels and BMD was characterized by a seemingly L-shaped curve in adolescents aged 12 to 15 years. The results of sensitivity analysis were consistent with the above analysis results. These findings indicate the negative association between urinary nitrate and BMD in adolescents. The findings also underscore the significance of nitrates exposure levels in identifying individuals at risk for low BMD.

## 1. Introduction

Osteoporosis (OP) is a systemic metabolic bone disorder defined by reduced bone mass, decreased bone mineral density (BMD), and an elevated risk of fractures.^[1]^ OP is prevalent among the elderly, particularly in postmenopausal women.^[2]^ Numerous studies have demonstrated a rising prevalence of OP, leading to significant implications for global health systems and socio-economic conditions.^[3–5]^ Adolescence represents a critical period for skeletal development, as the bone mineral content accumulated during this phase is significantly correlated with the future risk of OP.^[6]^ As bone mass naturally declines with age, the risk of developing OP progressively increases. In recent years, numerous studies have reported cases of OP in adolescents, at increasingly younger ages.^[7–9]^ BMD is a key diagnostic marker for OP. Therefore, clarifying the factors affecting BMD in adolescents is crucial for preventing OP.

Increasing evidence suggests that BMD is influenced by environmental exposures, including nitrate, volatile organic compounds, bisphenol A, perfluorinated and polyfluorinated alkyl substances, and phthalates.^[6,10–13]^ Nitrate is a widespread environmental contaminant, commonly present in soil, water, and processed foods.^[14]^ Many studies have identified the harmful effects of nitrate on human health. Research has indicated that elevated nitrate intake may be associated with an increased risk of developing gastric cancer.^[15]^ Long-term consumption of excessive nitrate levels has also been associated with an increased risk of developing type 2 diabetes, indicating a potential impact on the endocrine system.^[16]^ Zhang et al demonstrated the impact of nitrate in fine particulate matter on respiratory health, revealing that nitrate aerosols were linked to an elevated incidence of respiratory diseases.^[17]^ Several studies have indicated that nitrates exposure may impair thyroid function, potentially influencing bone metabolism and subsequently altering BMD.^[13,18–20]^ Furthermore, adolescents are more vulnerable to environmental pollutants compared to adults.^[6]^ It is noteworthy that the effect of nitrate on bone metabolism may exhibit a bidirectional regulatory role. Some studies suggest that under specific circumstances, nitrate may exert positive effects on skeletal health via the nitric oxide (NO) signaling pathway,^[21,22]^ indicating that the relationship between nitrates exposure and BMD may be more complex than a simple linear association. Urinary nitrate levels have been shown to serve as a biomarker for environmental exposure and can be used to assess the relationship between nitrates exposure and health outcomes.^[23]^ However, the association between nitrates exposure and BMD in adolescents remains unexplored.

Thus, utilizing data from the National Health and Nutrition Examination Survey (NHANES) database, the present study examines the relationship between urinary nitrate levels and BMD in adolescents aged 12 to 19, laying a preliminary scientific foundation for future research on OP and low BMD prevention.

## 2. Materials and methods

### 2.1. Study design and data sources

**Figure 1.**
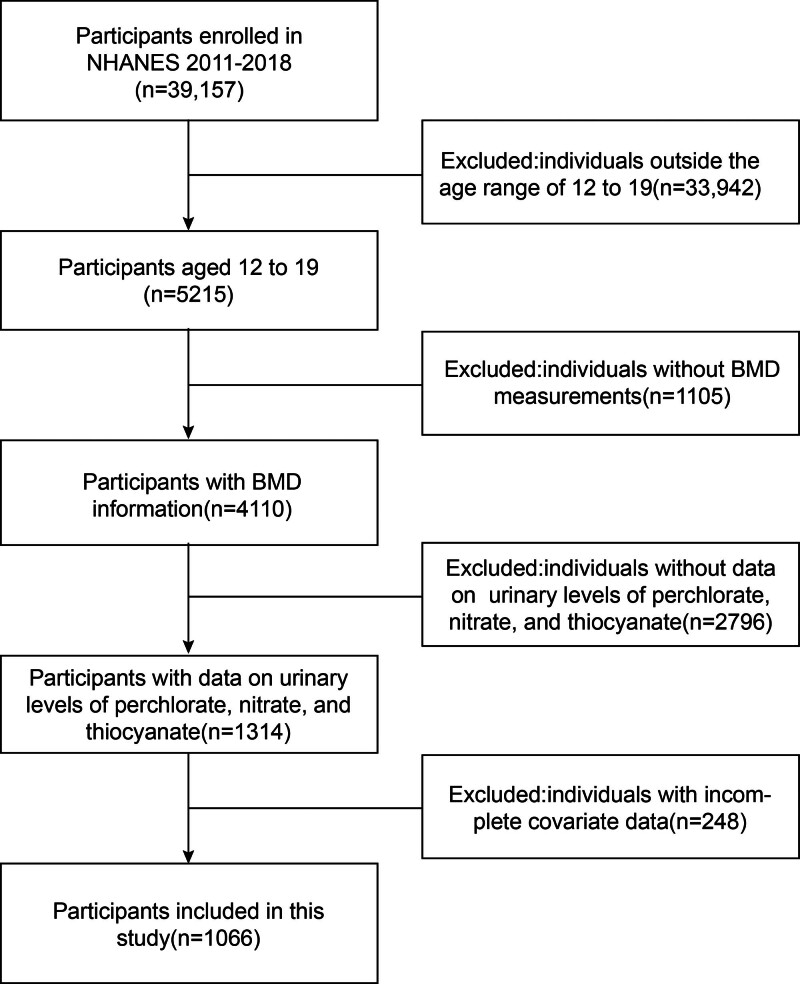
Flowchart for inclusion of participants from the NHANES 2011 to 2018. BMD = bone mineral density, n = number of participants, NHANES = National Health and Nutrition Examination Survey.

In order to evaluate the health and nutritional status of Americans, NHANES collected data via questionnaires, examinations, and laboratory measures spanning a wide range of health-related indicators, nutrient intake, and lifestyles. The data are both representative and reliable, extensively utilized in public health research and illness prevention. The NHANES data used in this study consist of completely public, de-identified data. Individual researchers using this dataset are not required to submit for Institutional Review Board approval. All original data collection had been approved by the NHANES Ethics Review Board. Data on 39,157 participants were extracted from the NHANES 2011 to 2018 database. Ultimately, 1066 participants were included in the analysis after excluding individuals outside the age range of 12 to 19 years (n = 33,942) and those with missing data on BMD (n = 1105), urinary nitrate (n = 2796), and relevant covariates (n = 248) (Fig. 1). The studies involving humans were approved by NHANES Ethics Review Committee.

### 2.2. Measurement of urinary nitrates

Urinary nitrates levels were measured using ion chromatography coupled with electrospray tandem mass spectrometry. Chromatographic separation was conducted with an Ion Pac AS16 column. The eluent from the column was ionized via an electrospray interface, generating negative ions, which were subsequently transferred to the mass spectrometer for analysis. Further methodological details can be found at https://wwwn.cdc.gov/nchs/nhanes/.

### 2.3. Examination of BMD

BMD was measured using dual-energy X-ray absorptiometry. Trained technicians performed scans of participants’ bones and soft tissues using Hologic Discovery Model A densitometers (Hologic, Inc., Bedford, MA) equipped with Apex 3.2 software. Experts ensured data quality throughout both the collection and processing phases. Detailed information is available in the Body Composition Procedures Manual on the NHANES website. Research had shown that the total body and lumbar spine are the preferred locations for measuring bone density in children and older adults.^[24]^ So, lumbar spine BMD and total BMD were designated as the dependent variables in this study.

### 2.4. Covariates

Drawing from the published literature,^[25–29]^ we identified relevant covariates to minimize potential confounding effects on the results, including sex, age, race, poverty-to-income ratio (PIR), body mass index (BMI), serum calcium, serum phosphorus, serum total protein, serum 25-hydroxyvitamin D_3_ (25(OH)D_3_), and urinary creatinine. For stratification, age was categorized into 2 groups (12–15 years and 16–19 years).^[26]^ The PIR was classified into 3 groups: low income (PIR < 1.3), middle income (PIR ≥ 1.3 and < 3.5), and high income (PIR ≥ 3.5).^[30]^ Race was categorized as non-Hispanic White, non-Hispanic Black, Hispanic, and Other.

## 3. Statistical analysis

R software (Version 4.3.0, R Foundation for Statistical Computing, Vienna, Austria) and Empower software (X&Y Solutions, Inc., Boston, MA) running on R software were utilized for all statistical analyses in this study. In all statistical analyses, a *P* value  < .05 was deemed statistically significant. By using weights, the complex multistage, stratified sampling strategy of NHANES can be taken into account statistically. Continuous variables were expressed as weighted means ± standard deviations, while categorical variables were presented as frequencies with weighted percentages.

To address potential biases arising from the multistage sampling design, we employed the WTSAF2YR-Fasting Subsample 2-Year MEC Weight for estimation correction. Following NHANES analytical guidelines, we calculated composite weights (MTMEC8YR) by proportionally allocating subsample weights across 4 survey cycles using the formula MTMEC8YR = (1/4) × WTMEC2YR, where WTMEC2YR represents the biennial subsample weight for individual survey phases.^[31]^ Urinary nitrates levels were adjusted for urinary creatinine concentration to account for the effects of urine dilution. We adjusted the urinary nitrates concentration using the formula Concentration _CR normalized_ = Concentration _specimen_ × (CR _reference_)/ (CR _specimen_).^[32]^ CR _reference_ is the average creatinine concentration and CR _specimen_ is the actual creatinine concentration (mg/dL).

Due to the skewed distribution of nitrate, creatinine-adjusted urinary nitrate levels were log-transformed using the natural logarithm. The nitrate levels were then categorized into quartiles as follows: < 10.663 ng/mL (Q1), 10.663 to 10.902 ng/mL (Q2), 10.903 to 11.172 ng/mL (Q3), and > 11.172 ng/mL (Q4). Weighted multivariate logistic and linear regression analyses were used to evaluate the association between urinary nitrate levels and BMD. 3 models were developed for the analysis. Model 1 was adjusted for age, sex, race, and urinary creatinine; Model 2 was further adjusted for BMI and PIR; Model 3 included additional adjustments for serum calcium, serum phosphorus, serum total protein, and serum 25(OH)D_3_. Smooth curve fitting adjusted model 3. Stratified analyses and interaction tests were subsequently performed to investigate the modifying effects of age, sex, race, and PIR on the association of urinary nitrate levels with BMD. Additionally, smoothed curve fitting and threshold effect models was employed to explore potential nonlinear relationships.

## 4. Results

### 4.1. Baseline population characteristics

The study included 1066 participants, with a mean age of 15.48 ± 2.20 years, 51.26% of whom were male and 55.56% were non-Hispanic White. Significant differences in age, race, BMI, serum calcium, serum phosphorus, serum total protein, serum 25(OH)D_3_, urinary creatinine, lumbar spine BMD, and total BMD were observed across the urinary nitrate quartiles (*P* < .05). BMI, serum phosphorus, and serum 25(OH)D_3_ levels were higher in the Q4 group compared to the Q1 group. Conversely, participants in the Q4 group were younger and exhibited lower levels of serum calcium, serum total protein, urinary creatinine, lumbar spine BMD, and total BMD. (*P* < .05, Table 1)

**Table 1:** Weighted characteristics of participants according to creatinine-adjusted and in-transformed nitrate.

Characteristic	Total	Q1	Q2	Q3		Q4		*P*
		N = 267	N = 266		N = 266		N = 267	
Age (yr)	15.48 ± 2.20	16.39 ± 2.01	15.51 ± 2.15		15.04 ± 2.13		15.12 ± 2.25	< .001
Sex								.101
Male	549 (51.26)	153 (57.49)	139 (52.17)		126 (47.26		131 (49.10)	
Female	517 (48.74)	114 (42.51)	127 (47.83)		140 (52.74		136 (50.90)	
Race								< .001
Non-Hispanic White	487 (55.56)	119 (44.68)	136 (50.95)		66 (62.48)		166 (62.22)	
Non-Hispanic Black	130 (11.82)	64 (23.94)	33 (12.52)		23 (8.63)		10 (3.82)	
Hispanic	234 (21.94)	49 (18.40)	74 (27.76)		54 (20.42)		57 (21.23)	
Other	115 (10.68)	35 (12.98)	23 (8.76)		23 (8.47)		34 (12.73)	
PIR								.136
Low income	240 (22.47)	70 (26.31)	58 (21.61)		51 (19.06)		63 (23.43)	
Middle income	526 (49.40)	137 (51.32)	121 (45.60)		135 (50.93)		132 (49.65)	
High income	300 (28.13)	60 (22.36)	87 (32.80)		80 (30.01)		72 (26.92)	
BMI (kg/m_2_)	24.01 ± 6.21	25.01 ± 6.36	23.83 ± 5.52		23.52 ± 6.07		23.83 ± 6.71	.035
Total calcium (mg/dL)	9.60 ± 0.28	9.64 ± 0.29	9.60 ± 0.27		9.59 ± 0.27		9.57 ± 0.29	.047
Phosphorus (mg/dL)	4.28 ± 0.66	4.15 ± 0.59	4.19 ± 0.65		4.37 ± 0.68		4.38 ± 0.69	< .001
Total protein (g/dL)	7.24 ± 0.40	7.31 ± 0.37	7.25 ± 0.41		7.23 ± 0.40		7.20 ± 0.41	< .001
Urinary creatinine (mg/dl)	134.53 ± 82.76	170.50 ± 102.65	141.63 ± 79.55		115.44 ± 65.32		116.20 ± 69.85	< .001
25(OH)D_3_ (nmol/L)	62.64 ± 23.72	58.33 ± 21.86	62.39 ± 23.78		64.76 ± 25.16		64.45 ± 23.17	.007
Lumbar spine BMD (g/cm^2^)	0.97 ± 0.15	1.04 ± 0.15	0.99 ± 0.14		0.94 ± 0.15		0.93 ± 0.15	< .001
Total BMD (g/cm^2^)	1.03 ± 0.12	1.09 ± 0.11	1.04 ± 0.11		1.01 ± 0.12		1.00 ± 0.12	< .001

Data are displayed as mean ± SD or N (%).

25(OH)D_3_ = serum 25-hydroxyvitamin D_3_, BMD = bone mineral density, BMI = body mass index, N = number of participants, PIR = poverty-to-income ratio, SD = standard deviation.

### 4.2. Association between nitrate levels and BMD

Table 2 presents the association between urinary nitrate levels and BMD, indicating that higher urinary nitrate levels were associated with lower BMD. In the partially adjusted models (Models 1 and 2), a significant negative correlation between urinary nitrate levels and BMD was observed. In the fully adjusted model (Model 3), this negative correlation persisted across multiple skeletal sites. Both lumbar spine BMD (β = −0.036, 95% confidence interval [CI]: −0.054, −0.019) and total BMD (β = −0.027, 95% CI: −0.040, −0.014) were negatively correlated with urinary nitrate levels. This suggests that for every unit increase in urinary nitrate, lumbar spine BMD decreased by 0.036 units, and total BMD decreased by 0.027 units.

**Table 2:** Association between nitrate levels and BMD.

Variable	Model 1	Model 2	Model 3
	β (95% CI)	β (95% CI)	β (95% CI)
Lumbar spine BMD			
Continuous variable	−0.042 (−0.060, −0.023)***	−0.040 (−0.058, −0.022)***	−0.036 (−0.054, −0.019)***
Categorical variable			
Q1	Reference	Reference	Reference
Q2	−0.012 (−0.034, 0.010)	−0.010 (−0.032, 0.011)	−0.014 (−0.036, 0.007)
Q3	−0.045 (−0.067, −0.023)***	−0.044 (−0.066, −0.022)***	−0.042 (−0.064, −0.020)***
Q4	−0.057 (−0.080, −0.034)***	−0.057 (−0.079, −0.035)***	−0.054 (−0.076, −0.032)***
Total BMD			
Continuous variable	−0.033 (−0.047, −0.019)***	−0.030 (−0.043, −0.016)***	−0.027 (−0.040, −0.014)***
Categorical variable			
Q1	Reference	Reference	Reference
Q2	−0.014 (−0.032, 0.003)	−0.013 (−0.029, 0.003)	−0.015 (−0.031, 0.001)
Q3	−0.028 (−0.045, −0.011)**	−0.027 (−0.043, −0.010)**	−0.025 (−0.041, −0.009)**
Q4	−0.043 (−0.060, −0.025)***	−0.043 (−0.059, −0.026)***	−0.040 (−0.057, −0.024)***

Model 1 adjusted for: age, sex, race, urinary creatinine; Model 2 adjusted for: age, sex, race, urinary creatinine, BMI, poverty income ratio; Model 3 adjusted for: age, sex, race, urinary creatinine, BMI, poverty income ratio, total calcium, phosphorus, total protein, 25(OH)D_3_.

25(OH)D_3_ = serum 25-hydroxyvitamin D_3_, BMD, bone mineral density, BMI = body mass index, CI = confidence interval.

* *P* < .05.

** *P* < .01.

*** *P* < .001.

The results further demonstrated a significant negative correlation between nitrate levels and BMD in groups Q3 and Q4 compared to group Q1, after categorizing nitrate levels. Additionally, the absolute β values progressively increased from Q3 to Q4, indicating that higher nitrate levels exerted a more pronounced negative effect on BMD.

**Figure 2.**
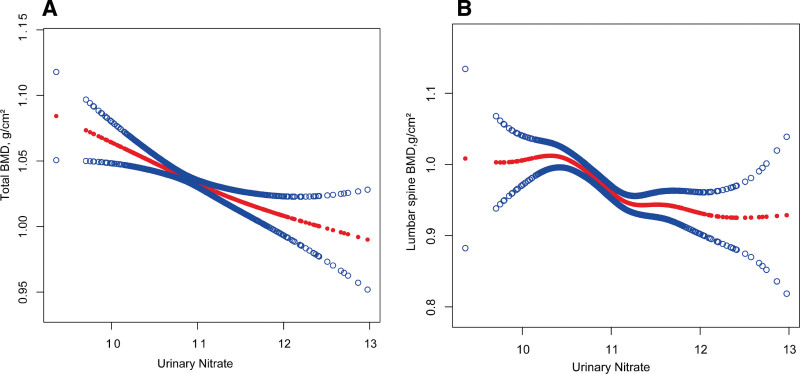
Dose-response association of urinary nitrate with (A) total BMD and (B) lumbar spine BMD. The solid red line represents the smooth curve fit between variables. Blue bands represent the 95% confidence interval from the fit. Model adjust for: age, sex, race, urinary creatinine, BMI, PIR, calcium, phosphorus, total protein, and 25(OH)D_3_. 25(OH)D3 = serum 25-hydroxyvitamin D_3_, BMD = bone mineral density, BMI = body mass index, PIR = poverty-to-income ratio.

In addition, smooth curve fitting also showed that urinary nitrate levels were negatively correlated with lumbar spine BMD (*P* < .001), and total BMD (*P* < .001) (Fig. 2).

## 5. Stratified analyses

**Figure 3.**
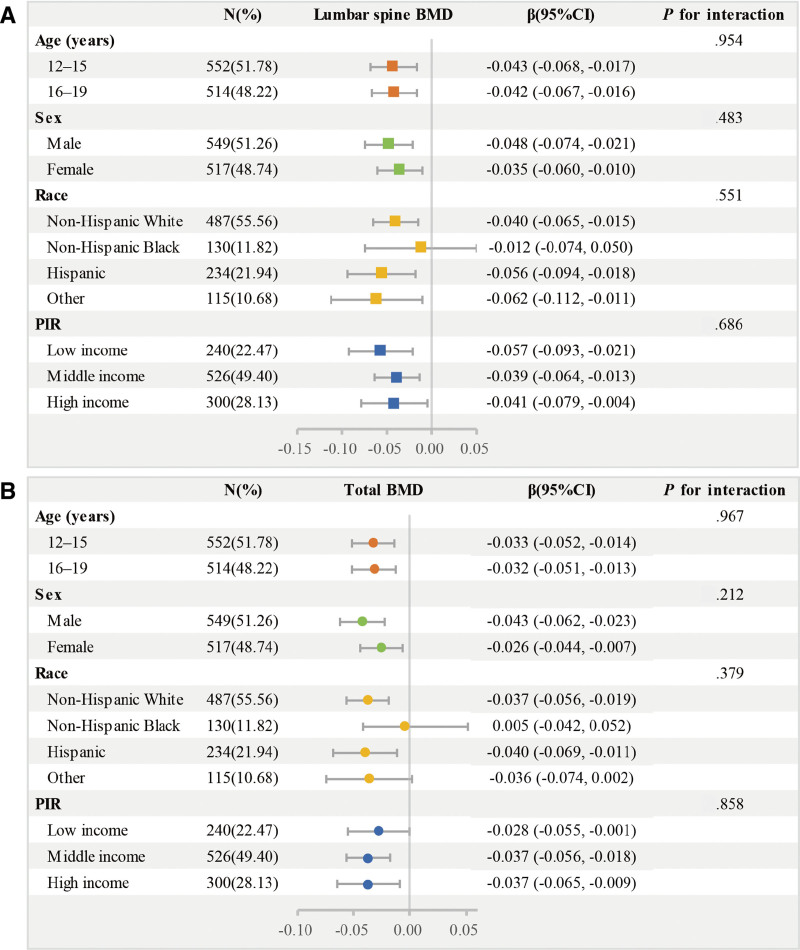
Subgroup analysis of the association of urinary nitrate with (A) lumbar spine BMD and (B) total BMD. The results were adjusted for all covariates except for the corresponding stratified variables. BMD = bone mineral density, CI = confidence interval, N = number of participants, PIR = poverty-to-income ratio.

We employed stratified weighted multivariate regression analysis to examine the association between nitrate levels and BMD in populations stratified by sex, age, race, and PIR. Nitrate levels negatively correlated with BMD in almost all stratified subgroups. However, among non-Hispanic Blacks, there was no significant correlation between nitrates exposure and BMD (*P* > .05). The interaction test indicated that the negative correlation between BMD and nitrate levels was not significantly affected by age, sex, race, or PIR (Fig. 3).

### 5.1. Analyses of the nonlinear relationship between nitrate and BMD

**Figure 4.**
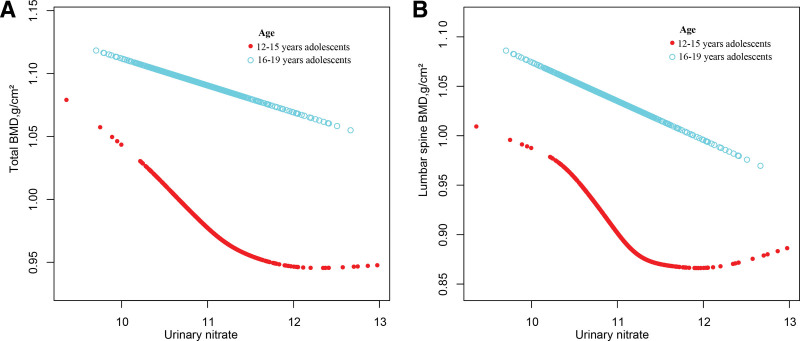
The association of urinary nitrate with (A) total BMDand (B) lumbar spine BMD. Sex, race, urinary creatinine, BMI, PIR, calcium, phosphorus, total protein, and 25(OH)D_3_ were adjusted. 25(OH)D_3_ = serum 25-hydroxyvitamin D_3_, BMD = bone mineral density, BMI = body mass index, PIR = poverty-to-income ratio.

To investigate the nonlinear relationship between nitrate and BMD, saturation threshold analysis and smooth curve fitting were employed. Adjusted smooth curve fitting indicated that in adolescents aged 12 to 15 years, lumbar spine BMD levels increased with rising nitrate levels until an inflection point (adjusted nitrate 11.145 ng/mL). Inflection point was similarly found between nitrate and total BMD (adjusted nitrate 11.144 ng/mL; Table 3; Figure 4). When nitrate levels exceeded or equaled the inflection point value, no significant correlation was observed between nitrate levels and BMD. Thus, the nonlinear association between nitrate and BMD was characterized by a seemingly L-shaped curve among adolescents aged 12 to 15 years.

**Table 3:** The threshold effect analysis of urinary nitrate on BMD in 12 to 15 subgroup (fitting by the 2-piecewise linear model).

	β(95CI), *P* value
Lumbar spine BMD	
Fitting by the standard linear model	−0.042 (−0.067, −0.017), .001
Fitting by the 2-piecewise linear model	
Inflection point	11.145
Urinary nitrate < 11.145	−0.125 (−0.170, −0.081), < .001
Urinary nitrate ≥ 11.145	0.031 (−0.010, 0.072), .137
*P* for Log-likelihood ratio	< .001
Total BMD	
Fitting by the standard linear model	−0.033 (−0.052, −0.014), .001
Fitting by the 2-piecewise linear model	
Inflection point	11.144
Urinary nitrate < 11.144	−0.097 (−0.130, −0.063), < .001
Urinary nitrate ≥ 11.144	0.022 (−0.008, 0.053), .155
*P* for Log-likelihood ratio	< .001

Sex, race, urinary creatinine, BMI, PIR, calcium, phosphorus, total protein, and 25(OH)D_3_ were adjusted.

25(OH)D_3_ = serum 25-hydroxyvitamin D_3_, BMD = bone mineral density, BMI = body mass index, CI = confidence interval, PIR = poverty-to-income ratio.

### 5.2. Sensitivity analysis

In the sensitivity analysis, we excluded extreme values where nitrate levels > 99% or < 1%. The results were consistent with the above analysis results, showing that nitrate levels were negatively correlated with lumbar spine BMD (β = −0.038, 95% CI: −0.058, −0.018; *P* < .001) and total BMD (β = −0.030, 95% CI: −0.045, −0.016; *P* < .001) (Table 4).

**Table 4:** Sensitivity analysis of the association of nitrate with BMD.

Variable	Model 1	Model 2	Model 3
	β (95% CI)	β (95% CI)	β (95% CI)
Lumbar spine BMD			
Continuous variable	−0.044 (−0.064, −0.024)***	−0.043 (−0.063, −0.023)***	−0.038 (−0.058, −0.018)***
Categorical variable			
Q1	Reference	Reference	Reference
Q2	−0.009 (−0.031, 0.013)	−0.007 (−0.029, 0.015)	−0.012 (−0.034, 0.009)
Q3	−0.042 (−0.065, −0.020)***	−0.042 (−0.064, −0.019)***	−0.040 (−0.062, −0.018)***
Q4	−0.054 (−0.076, −0.031)***	−0.054 (−0.076, −0.032)***	−0.050 (−0.073, −0.028)***
Total BMD			
Continuous variable	−0.036 (−0.051, −0.020)***	−0.034 (−0.049, −0.019)***	−0.030 (−0.045, −0.016)***
Categorical variable			
Q1	Reference	Reference	Reference
Q2	−0.016 (−0.033, 0.001)	−0.014 (−0.030, 0.003)	−0.017 (−0.033, −0.001)
Q3	−0.028 (−0.045, −0.010)**	−0.026 (−0.043, −0.010)**	−0.025 (−0.041, −0.009)**
Q4	−0.043 (−0.060, −0.025)***	−0.042 (−0.059, −0.026)***	−0.040 (−0.056, −0.024)***

Model 1 adjusted for: age, sex, race, urinary creatinine; Model 2 adjusted for: age, sex, race, urinary creatinine, BMI, PIR; Model 3 adjusted for: age, sex, race, urinary creatinine, BMI, PIR, total calcium, phosphorus, total protein, 25(OH)D_3_.

25(OH)D_3_ = serum 25-hydroxyvitamin D_3_, BMD = bone mineral density, BMI = body mass index, CI = confidence interval, PIR = poverty-to-income ratio.

* *P* < .05.

** *P* < .01.

*** *P* < .001.

## 6. Discussion

This study explored the association between nitrates exposure and BMD in adolescents, utilizing data from the NHANES 2011 to 2018 cohort. The results indicated that urinary nitrate levels were significantly and negatively correlated with lumbar spine BMD, and total BMD. This negative association persisted after adjusting for confounding factors. Through stratified analysis and smooth curve fitting, we observed a L-shaped relationship curve between nitrate levels and BMD in the 12 to 15 age group.

This study revealed a significant negative correlation between nitrate levels and BMD in adolescents, providing novel epidemiological evidence for the impact of environmental pollutants on skeletal development. Previous research by Wang et al demonstrated negative associations of urinary perchlorate, nitrate, and thiocyanate levels with BMD in general populations through mixed exposure effect models, with nitrate exhibiting the most pronounced contribution.^[13]^ Distinct from the broad population research, our investigation represents the first analysis focusing on adolescents, a critical period for bone development. Notably, multiple studies corroborate the link between nitrates exposure and BMD reduction,^[13,25,33]^ aligning with our findings. The precise mechanisms by which nitrate affects BMD remain incompletely understood and may be elucidated through the following approaches. Dietary intake and water consumption are the primary sources of human exposure to nitrates, which are pervasive in the environment. Epidemiological data indicate that dietary nitrate consumption originates predominantly from vegetables.^[34]^ Mechanistically, dietary nitrate is converted to NO by oral bacteria and various reductases in the body, thereby enhancing NO bioavailability via the nitrate-nitrite-NO pathway.^[35–37]^ NO exerts a biphasic modulatory effect on the activity of osteoclasts and osteoblasts.^[38–40]^ Consequently, nitrate may modulate the function of osteoclasts and osteoblasts, potentially leading to alterations in BMD. Alternatively, several studies have indicated that nitrate may disrupt thyroid function and development. Xie et al demonstrated that nitrate exposure could interfere with the TH signaling pathway. They proposed that nitrate suppresses deiodinase and thyroid hormone transporter gene expression (SLC16A2 and SLCO1C), hypothesizing that nitrate-derived reactive NO may damage essential proteins, thereby affecting mRNA stability and transcription.^[19,41]^ Furthermore, nitrate competitively inhibits the sodium iodide symporter, reducing iodine uptake and subsequently impairing thyroid hormone synthesis.^[34]^ Such endocrine disruption may perturb bone metabolism via altered thyroid-stimulating hormone and thyroxine (T4) signaling.^[42,43]^ It should be noted that the relationship between nitrates exposure and BMD may not be straightforward. As suggested by some studies, nitrate may exert protective effects on skeletal health under certain conditions through NO-mediated mechanisms.^[21,22]^ This bidirectional regulatory characteristic highlights the need for cautious interpretation of epidemiological findings. Future research should focus on elucidating the specific mechanisms by which nitrates affect bone health across different exposure levels, durations, and individual characteristics.

Subgroup analyses stratified by age indicated that the effect of nitrate on BMD varied across different subgroups. Specifically, a threshold effect was observed in adolescents aged 12 to 15 years, while a substantial linear association was found in those aged 16 to 19 years. Adolescence is a period of significant physical development, including changes in body composition, fluctuations in metabolism and hormones, maturation of organ systems, and the building of nutritional reserves, all of which may affect future health.^[44]^ The accelerated physical development and endocrine system transformations characteristic of adolescence, typically occurring at approximately 12 years in females and 14 years in males, may account for the threshold effect observed in the younger subgroup.^[45]^

The following are the strengths of this study. First, although studies of the relationship between urinary nitrate and BMD focused on adult populations, this is the first investigation into the association between nitrates exposure and BMD in adolescents. Second, the study’s large and weighted sample size enhances its representativeness. These findings will be instrumental for future investigations into the relationship between nitrates exposure and bone metabolism in adolescents. This study has certain limitations. First, due to the cross-sectional nature of the study, causal relationships between nitrates exposure and BMD cannot be established. Therefore, further research is needed to validate these findings and establish causality. Second, despite adjusting for several plausible confounders, the possibility of residual confounding cannot be entirely ruled out. Furthermore, this study is based on a multiethnic US population, whose genetic backgrounds, dietary habits, and environmental exposure profiles differ from those of Chinese and other specific populations. Therefore, caution should be exercised when extrapolating these findings to such groups. Future validation studies in diverse populations are warranted to confirm the generalizability of these associations. Finally, although several key covariates were adjusted for in this study based on previous literature in this field, we acknowledge that data on specific endocrine disorders (such as thyroid dysfunction, diabetes mellitus, and hypophosphatemic rickets) were not available for exclusion analysis. This represents a potential limitation of our study, as these conditions could potentially influence both nitrate metabolism and BMD. Future studies incorporating more comprehensive endocrine assessments would be valuable to confirm and extend our findings.

## 7. Conclusions

The findings revealed the negative correlation between BMD and nitrate levels in adolescents. The present findings underscore the significance of nitrates exposure levels in identifying individuals at risk for low BMD.

## Acknowledgments

We thank the staff and survey respondents who participated in the National Health and Nutritional Examination Survey.

## Author contributions

**Conceptualization:** Shuo Duan, Shuaiwei Li, Yisi Wang.

**Methodology:** Shuaishuai Wang, Zhiyang Liu, Minglei Zhang.

**Software:** Shuo Duan.

**Validation:** Shuaishuai Wang, Minglei Zhang.

**Visualization:** Shuo Duan.

**Writing – original draft:** Shuo Duan.

**Writing – review & editing:** Shuo Duan, Minglei Zhang.
